# A Proposal

**Published:** 1801-11

**Authors:** 


*To the Editors of the Medical and Phyſical Journal*,

### A PROPOSAL.

VACCINE Inoculation, diſcovered by Dr. Jenner, is the moſt fortunate invention for the benefit of mankind that ever was accompliſhed. The ſmall-pox, for more than a thouſand years, has deſtroyed a much greater proportion of the human race than any other peſtilence or diſeaſe that ever exiſted. This improved method of inoculation, by its ſafety from danger, by its quality of preventing the caſual ſmall-pox, and of communicating the diſtemper by contact only, and not by miaſms, is capable of preſerving more lives and preventing more miſery than any man, from either ſuperior power, art, or knowledge, had ever before poſſeſſed. By practicable regulations to protect the unthinking multitude from the caſual ſmall-pox, this loathſome and mortal diſtemper might ſoon be exterminated from the Britiſh iſlands, and from all civilized nations.

With an ardour and energy highly honourable to England, and to the medical character, this happy diſcovery, in leſs than three years, has been communicated to every enlightened people, even the remoteſt in the world.

The inventor of this moſt ſalutary art ought to receive a public reward, both as a token of its great importance and of Britiſh gratitude. To the lateſt poſterity it will be regarded as a national honour. To neglect or delay ſo juſt a remuneration would be a public diſgrace.

Should not a memorial, ſigned by the phyſicians, ſurgeons, and apothecaries of ſuch towns as approved the meaſure, be preſented to Parliament, humbly to requeſt that ſuch a reward as in their wiſdom may be thought proper, may be beſtowed upon Dr. Jenner for his highly meritorious ſervices? Would not ſuch a petition come moſt properly and honourably from the medical faculty? Would it not receive their general approbation and ſignature? Would not ſuch teſtimony have great influence both to obtain the reward, and to increaſe the practice of Vaccination?

*Bath, Sept.* 27, 1801. I. H.

This Hint is ſuggeſted by a perſon who is not perſonally known to Dr. Jenner.

